# Cognitive load predicts point-of-care ultrasound simulator performance

**DOI:** 10.1007/s40037-017-0392-7

**Published:** 2018-01-05

**Authors:** Sara Aldekhyl, Rodrigo B. Cavalcanti, Laura M. Naismith

**Affiliations:** 1 Department of Medicine, University of Toronto, Toronto, Ontario Canada; 2 Ho Ping Kong Centre for Excellence in Education and Practice, University Health Network, Toronto, Ontario Canada; 3 Centre for Addiction and Mental Health, Toronto, Ontario Canada

**Keywords:** Cognitive load theory, Simulation-based assessment, Point-of-care ultrasound

## Abstract

**Introduction:**

The ability to maintain good performance with low cognitive load is an important marker of expertise. Incorporating cognitive load measurements in the context of simulation training may help to inform judgements of competence. This exploratory study investigated relationships between demographic markers of expertise, cognitive load measures, and simulator performance in the context of point-of-care ultrasonography.

**Methods:**

Twenty-nine medical trainees and clinicians at the University of Toronto with a range of clinical ultrasound experience were recruited. Participants answered a demographic questionnaire then used an ultrasound simulator to perform targeted scanning tasks based on clinical vignettes. Participants were scored on their ability to both acquire and interpret ultrasound images. Cognitive load measures included participant self-report, eye-based physiological indices, and behavioural measures. Data were analyzed using a multilevel linear modelling approach, wherein observations were clustered by participants.

**Results:**

Experienced participants outperformed novice participants on ultrasound image acquisition. Ultrasound image interpretation was comparable between the two groups. Ultrasound image acquisition performance was predicted by level of training, prior ultrasound training, and cognitive load. There was significant convergence between cognitive load measurement techniques. A marginal model of ultrasound image acquisition performance including prior ultrasound training and cognitive load as fixed effects provided the best overall fit for the observed data.

**Discussion:**

In this proof-of-principle study, the combination of demographic and cognitive load measures provided more sensitive metrics to predict ultrasound simulator performance. Performance assessments which include cognitive load can help differentiate between levels of expertise in simulation environments, and may serve as better predictors of skill transfer to clinical practice.

**Electronic supplementary material:**

The online version of this article (10.1007/s40037-017-0392-7) contains supplementary material, which is available to authorized users.

## What this paper adds

High cognitive load during simulation training is associated with impaired learning and incomplete skill transfer to clinical practice. Measuring cognitive load can thus help to identify individuals whose skills are not fully consolidated. Using cognitive load measures in this way requires a higher standard of validity evidence than currently exists in the literature. Using point-of-care ultrasonography as a model, this study provides a proof-of-principle for how multiple cognitive load measures can be incorporated to strengthen validity claims. Across the expertise continuum, ultrasound simulator performance was most sensitively predicted by a combination of prior ultrasound training and cognitive load.

## Introduction

Simulation-based surgical and procedural skills training offers the ability to ensure patient safety while providing a standardized learning environment that is useful for both trainees and their clinical teachers. While the benefits of simulation-based training can exceed those of traditional clinical education for the acquisition of specific skills [1], evidence for inferring clinical competence on the basis of simulation training alone remains limited [2, 3]. Though commonly used, time and error-based metrics have shown to be insufficient for predicting transfer from the simulation environment to clinical practice [4, 5]. Establishing rigorous criteria to make judgements about trainee competence represents a key ongoing priority in simulation research [6].

Trainees’ ability to learn complex skills may depend on the level of cognitive load they experience during simulation training. Cognitive load refers to the degree to which a learner’s limited working memory is occupied during a learning task [7]. Working memory is used to process new information from the instructional environment and encode it into long-term memory in organizational structures known as schemas. Cognitive load theory proposes that an instructional design contributes to two principal types of cognitive load: intrinsic and extraneous [8]. Intrinsic load represents the working memory resources required to complete the learning task, and is influenced by both the element interactivity of the task (i. e. the number of essential task elements that must be processed simultaneously) and the sophistication of the learner’s existing schemas [7]. Tasks with a greater number of interacting elements are associated with higher levels of intrinsic load. For example, performing a procedure on a task trainer while simultaneously communicating with a standardized patient has a higher intrinsic load than performing the procedure alone [9]. As expertise develops, task schemas become automated and occupy less space in working memory [10]. This implies that achieving equivalent performance on a task will be associated with less intrinsic load for an expert than for a novice. Extraneous load refers to the working memory resources allocated towards processing aspects of the instructional design that do not contribute to task performance. Extraneous load can arise from multiple sources including how the task is presented [8], the physical environment [11], and the learner’s emotions [12].

Cognitive load theory focuses on how to design instruction such that maximal working memory resources are devoted towards increasingly expert-like schema construction. According to this theory, learning can be impaired if a learner’s total cognitive load (intrinsic load + extraneous load) exceeds his or her working memory capacity [7]. Empirically, simulation training under high cognitive load conditions has been associated with impaired learning and incomplete skill transfer to other simulated tasks and environments [12, 13]. Measuring cognitive load during simulation training may thus help to identify individuals whose skills are not fully consolidated [4, 13]. Using cognitive load measurement to inform judgements of competence demands a high standard of validity evidence [14]. To date, the measurement of cognitive load in simulation settings has been reliant on retrospective, self-reported data [14], with some preliminary validity evidence for more objective measures such as secondary tasks [15] and physiological indices [16].

This exploratory, proof-of-principle study investigated the correspondence of multiple cognitive load measures within the specific context of simulation-based point-of-care ultrasonography (POCUS). POCUS in internal medicine involves limited examinations that are performed at the bedside in support of specific diagnostic or procedural aims [17]. In contrast to the comprehensive training required by radiologists and sonographers, there is currently little consensus as to the training and competency requirements for internists to use POCUS safely in the clinical environment [18]. While preliminary evidence suggests that training with a high fidelity simulation is effective in preparing trainees for simulation-based POCUS assessment [19], there is little evidence to support direct clinical transfer. In this study, we set out to understand whether cognitive load measures could inform our understanding of the correspondence between clinical expertise and performance on simulated POCUS tasks. Specifically we examined relationships between: a) demographic markers of clinical expertise (i. e., level of training, prior clinical point-of-care ultrasound experience); b) measures of cognitive load; and c) ultrasound simulator performance.

## Methods

### Participants

A sample (*n* = 29) of trainees and clinicians affiliated with the University of Toronto and currently practising in internal medicine, emergency medicine or intensive care was recruited via emails sent by a research coordinator. Participants included medical students (*n* = 3), junior residents (*n* = 4), senior residents (*n* = 14), and staff physicians (*n* = 8) with varying levels of clinical experience with point-of-care ultrasound. While this was primarily a convenience sample based on participant availability, we purposefully selected for variation in clinical experience. The majority of participants (24, 83%) were primarily affiliated with general internal medicine. This study was approved by the Research Ethics Board at the University of Toronto and participants were offered a $10 gift card as compensation for their time.

### Procedure

This study adopted a single-group repeated measures design. Participants were tested individually in a simulation laboratory. After obtaining written consent, all participants completed (1) a demographic questionnaire to assess their level of prior ultrasound training, clinical point-of-care ultrasound experience, and prior simulator exposure; and (2) a brief, paper-based structure-labelling exercise of a clinical ultrasound image to assess baseline ultrasound interpretation skills. Thereafter, each participant put on a pair of head mounted eye tracking glasses (SensoMotoric Instruments GmbH, Teltow) and was led through a 3-point calibration exercise according to manufacturer instructions. We were unable to obtain eye tracking data for one participant due to a technical malfunction with the eye tracking glasses.

Binocular eye data were collected continuously at a frequency of 30 Hz. Video and audio recordings of each participant were obtained through two cameras (one mounted on the eye tracker and another mounted on a stand). Ultrasound scanning tasks were performed using the Vimedix Ultrasound Simulator (CAE Healthcare, Saint-Laurent), which includes a mannequin and a haptic probe. The diagnostic labels of the pre-programmed pathologies were concealed on the ultrasound monitor (stealth mode). To conclude the data collection protocol, all participants completed a feedback questionnaire about their perceptions of the realism of the ultrasound simulator (0 = not realistic; 4 = extremely realistic) and their opinion on whether simulators should be a standard part of an ultrasound curriculum. The average time required to complete the data collection protocol was 25 min.

### Outcome measures

#### Ultrasound image acquisition

Our primary performance outcome was participants’ ability to acquire a clinically interpretable image using the ultrasound simulator. Participants were presented with six scenarios of variable difficulty, each of which prompted them to scan the mannequin for a pre-programmed relevant pathology. The scenarios were designed to assess ultrasound skills pertinent to internal medicine training and were finalized following a series of pilot studies. For example, easy scenarios required visualization of a major organ such as the liver or kidney. Easy scenarios were low in element interactivity in that the essential task elements could be processed sequentially: placing the ultrasound probe on the mannequin at a known landmark position, recognizing a defined structure with typical anatomy, and perhaps adjusting the probe position slightly if the image was unclear. More difficult scenarios required participants to scan for a pathological finding such as a small pocket of ascites or a pleural effusion. These scenarios were higher in element interactivity, in that the participant was required to move the probe over a larger area of the mannequin while simultaneously comparing the acquired image against schemas for both typical and atypical anatomy. During the study, the task stems were projected on an adjacent laptop computer screen in a standardized but random order in terms of difficulty to minimize any carryover effect secondary to practice and learning. Participants were asked to verbalize relevant findings and commit to a diagnosis within the 2 min allocated for each task. Image acquisition quality was scored in real-time by a single rater (SA) utilizing a 3-point checklist (0 = could not visualize; 1 = acceptable view; 2 = excellent view). A second rater (RBC) scored video recordings of a randomly selected subset of 8 participants, representing 28% of the total data set.

#### Ultrasound image interpretation

To ensure that any performance deficits in ultrasound image acquisition were not due to lack of anatomical knowledge, we independently assessed participants’ ability to interpret ultrasound images. Following each of the six scenarios, we displayed a clear image of the target pathology that we had previously acquired from the ultrasound simulator and asked participants to verbally identify the relevant anatomical structures. The number of structures for each scenario ranged from 1 to 4 and each structure was scored as either correct or incorrect. The total image interpretation score for each task ranged from 0 to 1 and represented the average number of correct structure identifications.

#### Cognitive load

We used two self-report questionnaires to measure the subjective total cognitive load of each scenario: the Paas scale [20] and the NASA Task Load Index (NASA-TLX) [21]. The Paas scale is a single-item measure of invested mental effort (1 = very, very low mental effort; 9 = very, very high mental effort). The NASA-TLX provides an overall workload score (range: 0–120) that is calculated as the sum of six 20-point subscales: mental demand, physical demand, temporal demand, performance, effort and frustration. Participants completed both questionnaires immediately after each scenario. Physiological measures of total cognitive load included left pupil diameter (mean and range) and blink rate [22]. Based on pilot data, we also investigated whether total scanning time and the rate of gaze shifts between the haptic probe and the ultrasound monitor were associated with other cognitive load measurements.

### Data analysis

All data analyses were carried out in SPSS version 22 (IBM, Redmond). Statistical significance was interpreted as a *p*-value less than 0.05.

#### Demographic measures

The first quartile value (35) of the self-reported number of clinical point-of-care ultrasound procedures performed (range: 0–300) was used to classify participants into experienced (*n* = 8, ≥35 procedures) and novice (*n* = 21, <35 procedures) groups. Four-point ordinal scales were constructed for level of training (0 = medical student; 1 = junior resident; 2 = senior resident; 3 = staff) and prior ultrasound training (0 = none; 1 = informal; 2 = brief; 3 = extensive). Prior simulator exposure was scored as a dichotomous variable (0 = no; 1 = yes). Independent samples *t*-tests were used to compare mean levels of each demographic variable. We corrected for degrees of freedom when the Levene’s test for equality of variances resulted in a *p*-value less than 0.05 (i. e., variances were significantly different between groups).

#### Comparisons and predictors of ultrasound simulator performance

We used multilevel linear modelling (MLM) to analyze repeated measures data. MLM is an extension of multiple linear regression that can account for correlations between observations and errors as well as unbalanced designs and unequal variances between groups [23–25]. In MLM, the dependent variable is modelled as the sum of fixed effects of one or more independent variables, random effects owing to the particular sampling strategy employed, and errors. Our modelling approach included testing for both random slope and random intercept effects [26]. If random effects were not significant, we substituted marginal, also known as population-averaged, models that included scenario as a repeated effect [27].

We analyzed 4 of the 6 scenarios for performance differences between experienced and novice participants. We excluded the first scenario to minimize any performance or cognitive load effects associated with simulator familiarization and excluded an additional scenario due to the presence of a simulator artefact that was interpreted inconsistently across the participant group. Inter-rater reliability for ultrasound image acquisition performance was assessed based on the intraclass correlation coefficient (ICC, two-way random model, single measure, absolute agreement) and Cronbach’s alpha (α). We constructed separate models for ultrasound image acquisition and ultrasound image interpretation performance and tested the fixed effect of expertise, coded as a dichotomous variable (0 = novice; 1 = experienced). Each model contained 116 observations (29 participants × 4 tasks).

We then tested predictors of our primary outcome variable, ultrasound image acquisition performance. We first tested the fixed effects of individual predictors (i. e., level of training, prior ultrasound training, cognitive load) and then tested all possible combinations of predictors, using the likelihood ratio criterion to select the best fitting model. Using this criterion, a smaller value indicates a better fitting model [25].

#### Processing of eye tracking data

Following data collection, all data files from the eye tracker were imported into the BeGaze 3.5 software package (SensoMotoric Instruments GmbH, Teltow). To determine the precise start and end points of each scenario, we reviewed all videos in the Scan Path data view, which displays the gaze position of the participant plotted on a video of the scene. The start point of the task was interpreted as the first video frame in which the gaze position was superimposed on the ultrasound monitor after the participant placed the probe on the mannequin. The end point of the task was the moment after the participant stated his or her finding or abandoned the task. Both values were recorded in seconds to 3 decimal places. We subtracted the start time from the end time to obtain the total scanning time in seconds. The number of gaze shifts represented the number of separate instances the participant’s gaze fixated on the ultrasound monitor. We divided this number by the total scanning time in seconds and multiplied it by 60 to arrive at a value for gaze shift rate per minute.

For each scenario, a raw data file including a timestamp, left and right pupil diameter in mm, and type of event (i. e., fixation, saccade, or blink) for each eye measurement sample was exported from BeGaze in ASCII text format. These files were then imported into SPSS version 22 (IBM, Redmond). The timestamp value was converted to time in seconds and filtered according to the start and end times recorded for each task. Blink rate was recorded as the percentage of samples that were labelled as blinks by a proprietary algorithm in BeGaze. Following literature-based practices, we calculated means and standard deviations of samples labelled as fixations where the left pupil diameter was greater than 0 [28, 29]. Outlying samples greater than 3 standard deviations from the trial mean were removed and means and standard deviations were recalculated. Means, standard deviations and ranges for left pupil measurements were recorded for each scenario.

To verify that the eye tracking glasses could detect variations in pupil diameter related to changing mental demands, we performed an additional calibration task with 18 participants. While wearing the eye tracking glasses, participants were instructed to verbally respond to two multiplication questions administered in an easy-to-difficult sequence. Between question 1 (2 × 5) and question 2 (16 × 32), we noted an average increase in maximum pupil size of 0.53 mm, which was in line with expected variation [22].

#### Comparison of cognitive load measures

To test for agreement between the different cognitive load measures, we constructed a separate MLM model for each measure (i. e., NASA-TLX, pupil diameter mean, pupil diameter range, blink rate, total scanning time, gaze shift rate). In each model, the dependent variable was the Paas scale rating and the measure to be tested was modelled as a fixed effect. Observations from the four scenarios were clustered by participant. The NASA-TLX model was based on 116 observations (29 participants × 4 tasks), while the remainder of the models were based on 112 observations (28 participants × 4 tasks).

#### Participant feedback

An independent samples *t*-test was used to compare perceptions about the realism of the ultrasound simulator between the novice and experienced participants.

## Results

### Demographic measures

Experienced participants were significantly more advanced in their training and had completed a significantly greater number of clinical point-of-care ultrasound procedures (Table 1). These participants also tended to have higher levels of prior ultrasound training and simulator exposure, though comparisons did not reach levels of statistical significance. Experienced participants outperformed novices on the structure labelling pretest (*M*_Experienced_ = 0.97/1.00, *SD* = 0.09; *M*_Novice_ = 0.71/1.00, *SD* = 0.35; *t*_25.29_ = −3.11,* p* < 0.01, *d* = 1.28, 95% CI 0.41 to 2.16).

**Table 1:** Demographic characteristics of study participants

Demographic variable	Experienced (*n* = 8) mean ± SD	Novice (*n* = 21) mean ± SD	*t*-test comparisons			Effect size	
			*t*	df	*p*-value	d	95% CI
Level of training^a^	2.75 ± 0.46	1.62 ± 0.87	−3.49	27	<0.01	1.45	0.55 to 2.35
Prior ultrasound training^b^	2.25 ± 0.71	1.71 ± 0.64	−1.95	27	0.06	0.81	−0.03 to 1.65
Prior clinical point-of-care ultrasound procedures	107 ± 94	12 ± 11	−2.83	7.07^d^	0.03	0.99	0.13 to 1.84
Prior simulator exposure^c^	0.75 ± 0.46	0.43 ± 0.51	−1.63	13.85^d^	0.13	0.66	−0.18 to 1.49

*SD* standard deviation, *df* degrees of freedom, *d* effect size (Cohen’s d), *CI* confidence interval

^a^*0* medical student, *1* junior resident, *2* senior resident, *3* staff physician

^b^*0* none, *1* informal, *2* brief, *3* extensive

^c^*0* no, *1* yes

^d^*df* corrected to account for unequal variances

### Comparisons and predictors of ultrasound simulator performance

**Fig. 1:**
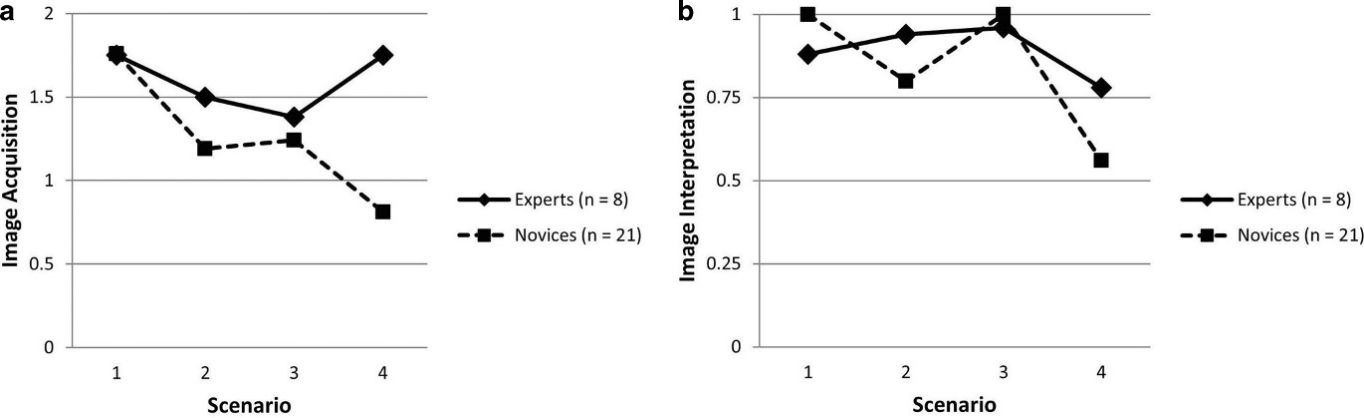
Comparisons of experienced and novice performance in: **a** ultrasound image acquisition; **b** ultrasound image interpretation. Scenarios: *1* right kidney, *2* large pericardial effusion, *3* fluid in Morison’s pouch, *4* moderate pleural effusion. Notes: Scenarios are ordered here according to expected difficulty, but were completed in a different order by participants. The y‑axes depict the full range of possible performance scores

Fig. 1 illustrates performance comparisons between the two groups across the 4 scenarios analyzed. Using marginal models with scenario included as a repeated effect, we found a significant fixed effect of expertise on ultrasound image acquisition performance. The marginal model included an intercept, *B* = 1.59, *SE* = 0.13, 95% CI 1.33 to 1.86, *t*_114_ = 11.90, *p* < 0.001, and a significant fixed effect of being classified a novice, *B* = −0.34, *SE* = 0.16, 95% CI −0.66 to −0.03,* t*_114_ = −2.18, *p* = 0.03. The average experienced participant score can be interpreted as the intercept estimate of 1.59 (out of a maximum score of 2) and the average novice score can be calculated as 1.25 (i. e., 1.59–0.34). The covariance estimate for repeated measures was significant, *B* = 0.57, *SE* = 0.08, 95% CI 0.44 to 0.74, Wald* Z* = 7.55, *p* < 0.001, supporting our choice of model. Inter-reliability for a single measure of image acquisition was acceptable, ICC_(2,1)_ = 0.61, 95% CI 0.34–0.79; α = 0.76. The model for image interpretation performance includes an intercept, *B* = 0.89, *SE* = 0.05, 95% CI 0.79 to 0.98, *t*_114_ = 18.70, *p* < 0.001, and a non-significant fixed effect for having a novice classification, *B* = −0.05, *SE* = 0.06, 95% CI −0.16 to 0.06,* t*_114_ = −0.87, *p* = 0.38. The covariance estimate for repeated measures was again significant, *B* = 0.07, *SE* = 0.01, 95% CI 0.06 to 0.09, Wald* Z* = 7.55, *p* < 0.001.

**Fig. 2:**
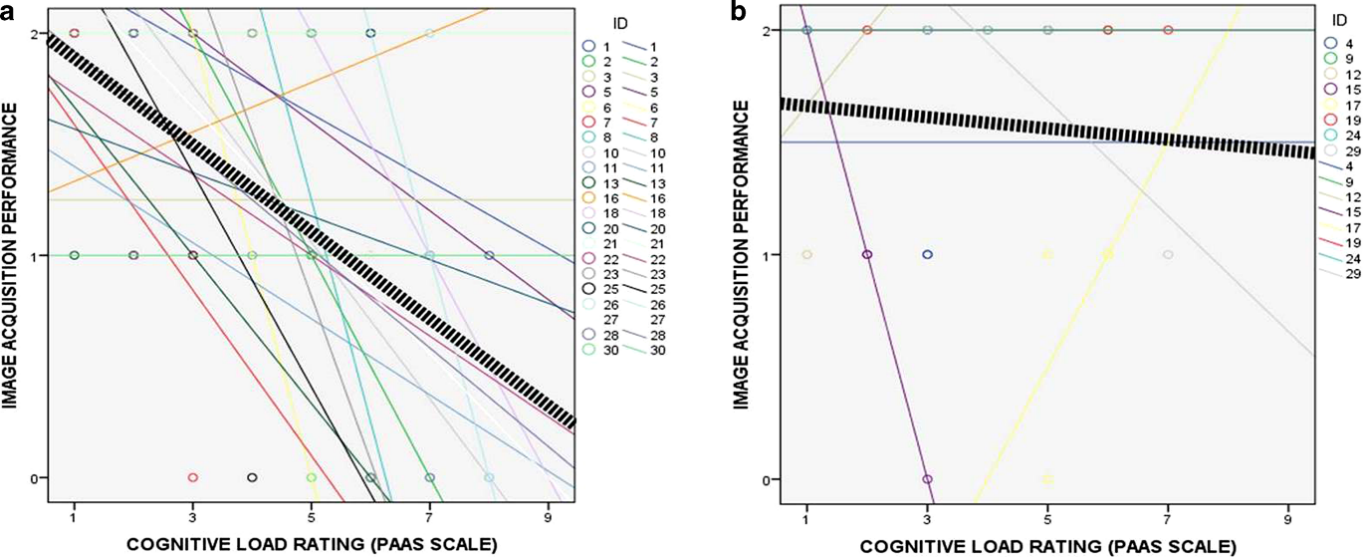
Relationships between cognitive load rating (Paas scale) and image acquisition performance for: **a** Novice participants (*n* = 21); **b** Experienced participants (*n* = 8)

When tested individually, level of training, prior ultrasound training, and cognitive load (Paas scale rating) all predicted ultrasound image acquisition performance on MLM analyses (see the Electronic Supplementary Material (ESM), Tables A‑1, A‑2 and A‑3). In particular, high cognitive load was associated with poor performance for novices, whereas experienced participants were able to maintain good performance even with a higher cognitive load (Fig. 2). After testing all possible combinations of predictors, a marginal model with prior ultrasound training and cognitive load as fixed effects and scenario as a repeated effect provided the overall best fit for the observed data (Table 2). The value of the likelihood ratio criterion for the model with both prior ultrasound training and cognitive load as predictors was 253.21, compared with a 263.74 for prior ultrasound training alone, and 255.79 for cognitive load alone.

**Table 2:** Parameter estimates for marginal model with ultrasound image acquisition as the dependent variable and prior ultrasound training and cognitive load (Paas scale rating) as predictor variables

Fixed effect	B (SE)	df	t-value	*p*-value	95% CI
Intercept	1.50 (0.27)	113	5.64	<0.01	0.97 to 2.02
Prior ultrasound training^a^	0.22 (0.10)	113	2.34	0.02	0.03 to 0.42
Cognitive load^b^	−0.14 (0.03)	113	−4.05	<0.01	−0.21 to −0.07
*Covariance parameter*	*V (SE)*	*Wald Z*	*p-value*		
Repeated measures	0.48 (0.06)	7.52	<0.01		

*B* regression coefficient, *SE* standard error, *df* degrees of freedom, *CI* confidence interval, *V* covariance

^a^*0* none, *1* informal, *2* brief, *3* extensive

^b^ range 1–9

### Comparison of cognitive load measures

We compared different types of cognitive load measures with the Paas scale to better understand what measures might be useful indicators of mental effort in simulation training. Using random intercept models, we found NASA-TLX rating, pupil diameter range, total scanning time, and gaze shift rate to be significant predictors of Paas scale rating (ESM, Table A-4). For example, the NASA-TLX model includes an intercept, *B* = 1.48, *SE* = 0.22, 95% CI 1.04 to 1.93, *t*_65.78_ = 6.65, *p* < 0.001, and a significant fixed effect for NASA-TLX rating, *B* = 0.067, *SE* = 0.004, 95% CI 0.060 to 0.075,* t*_112.89_ = 16.93, *p* < 0.001. A NASA-TLX rating of 100/120 would therefore correspond to a Paas scale rating of 8.18/9 (i. e., 1.48 + 0.067 × 100). The intercept variance estimate was significant, *B* = 0.59, *SE* = 0.20, 95% CI 0.30–1.15, Wald* Z* = 2.96, *p* = 0.003, supporting a random intercept model. We observed a negative relationship for gaze shift rate, with an intercept, *B* = 5.23, *SE* = 0.35, 95% CI 4.53 to 5.92, *t*_69.26_ = 14.91, *p* < 0.001, and a significant fixed effect, *B* = −0.14, *SE* = 0.03, 95% CI −0.19 to −0.08,* t*_97.94_ = −4.65, *p* < 0.001, suggesting that each additional gaze shift per minute was associated with a decreased Paas scale rating. Fixed effect estimates for pupil diameter mean and blink rate were not significant.

### Participant feedback

To explore the impact of performing in a simulated setting we sought participant feedback on realism of the exercise. Overall, participants reported the experience as moderately realistic (*M = *1.97/4, *SD =* 1.05). We found no significant differences between groups in the perception of similarity with real patient encounters (*M*_Experienced_ = 2.38/4, *SD* = 0.52; *M*_Novice_ = 1.86/4, *SD* = 1.01; *t*(27) = −1.37, *p* = 0.18, *d = *0.57, 95% CI −0.26 to 1.40). The majority of participants in both groups (85% novices, 100% experienced participants) felt that simulators should be incorporated into a point-of-care ultrasound curriculum.

## Discussion

In this study we explored the correspondence between multiple cognitive load measures in the context of simulation-based POCUS. We classified participants into either novice or experienced groups on the basis of their prior experience performing POCUS in a clinical setting. Experienced participants outperformed novices in simulator-based ultrasound image acquisition, while simulator-based ultrasound image interpretation was comparable between the two groups. By adopting a multilevel linear modelling approach, we found that simulation-based ultrasound image acquisition performance could be predicted across participant groups by a combination of prior ultrasound training and cognitive load.

Findings from this study contribute to the growing body of literature documenting a negative relationship between cognitive load and performance in simulation settings [14]. In previous studies of simulation training, trainees reporting high levels of cognitive load made more frequent errors and showed impaired transfer during simulation [12, 13]. Using cognitive load theory as an instructional design framework provides both a means to identify these high load situations, and specific guidance as to how they may be ameliorated. For example, intrinsic cognitive load may be reduced by segmenting tasks into manageable chunks and/or by providing additional pre-training opportunities while extraneous cognitive load may be minimized through the use of worked examples and/or by reducing the need for learners to split their attention between multiple sources of information [30]. Our previous work has demonstrated that a cognitive load approach to instructional design can be feasible even in the context of short training interventions [31].

As competency-based assessment becomes more widespread in medical training [32], it becomes increasingly important to distinguish between those individuals whose skills are fully consolidated and those who can only maintain good performance with significant effort [33]. In making competency judgements for individual trainees, we contend that the ability to transfer learning from a relatively controlled simulation environment to a more complex and unpredictable clinical environment requires trainees to be able to consistently perform well with low cognitive load. This indicates that trainees still have spare working memory capacity to respond to unexpected changes in a patient’s condition as well as distracting stimuli, both of which are common occurrences in the clinical environment. Incorporating cognitive load measures in simulation-based training and assessment can provide an objective and reliable standard for identifying which individuals are working close to the limit of their abilities in a simulated setting and thus may benefit from further training and/or remediation before progressing to unsupervised clinical practice [14, 34].

But which cognitive load measure should we use? Several cognitive load measures allow for the practical assessment of working memory usage in a simulated setting [35]. This study demonstrated good convergence between self-report, physiological, and behavioural measures of cognitive load. To our knowledge, this is the first study to demonstrate an association between an observed behaviour (gaze shift rate) and an established measure of cognitive load (Paas scale rating) in the context of medical simulation training. While gaze shift rate is specific to ultrasound simulation, observer ratings in general are advantageous in that they provide the means for unobtrusive monitoring of cognitive load. Further research is necessary to develop and validate observed measures of behaviour that are more widely applicable across simulation settings, such as breaches in sterility [36]. Such behavioural measures would provide another easily acquired variable for triangulating measurements of cognitive load, an important step in ensuring their validity [14]. Given the consequences associated with judgements of competence and the inherent limitations of the various measurement methods, the use of multiple converging measures may be necessary to establish the necessary evidence standard [35].

The convergence that we found between the Paas and NASA-TLX scales may be attributed to both instruments’ sensitivity to intrinsic load variations [15, 37–39], as the feasibility of measuring extraneous load via self-report has been repeatedly called into question [37, 38, 40]. Our data collection protocol was carefully designed to maximize variations in task-related intrinsic load and minimize variations in extraneous load. With respect to the intrinsic cognitive load required to complete the scenarios, we devoted considerable time to designing and piloting tasks to ensure that task difficulty was a direct function of ultrasound image acquisition complexity. We also primarily selected participants who had a common interest and/or specialization in general internal medicine. The comparable performance between novices and experienced participants in ultrasound image interpretation suggests that differences in prior knowledge did not represent a significant source of intrinsic load variation. Thus, all participants who were able to acquire the image had sufficient knowledge to be able to interpret it correctly. To minimize any environmental distractions that might contribute to extraneous cognitive load [40], we collected participant data in a quiet, darkened room. In addition, all scenarios were short, focused, and presented in the same format. The voluntary, low-stakes nature of the study and the positive feedback from participants on the realism of the simulator suggest that the impacts of assessment-related anxiety and fidelity on extraneous cognitive load were negligible [37].

Our results regarding eye-based physiological measures were somewhat conflicting. While pupil diameter range showed significant concordance with the Paas scale rating, we failed to observe this relationship for mean pupil diameter or blink rate. In our estimation, the capture and interpretation of physiological measures continues to be hindered by high levels of inter- and intra-individual variability [14, 35]. We were also unable to replicate the conditions of previous medical education studies that relied on fixed eye-trackers and tightly controlled light conditions [16]. While we were able to observe a change in pupil diameter with our arithmetic calibration task, this effect could not be detected with the ultrasound scanning tasks, wherein the participant was moving and the brightness of the ultrasound monitor was continually changing. This calls into question the feasibility of pupil-based measures of cognitive load in dynamic simulation contexts.

Our study has several limitations. As there is no objective standard for determining expertise in point-of-care ultrasound, we cannot definitively conclude that our experienced/novice classification generalizes beyond our current sample. We are also limited by our choice of performance measure. While we observed adequate inter-rater agreement [41, 42], results from our image acquisition rating scale are limited to our specific simulation context and therefore do not permit direct comparison with scales tested in a clinical setting [43]. Future studies incorporating multiple raters from the outset would permit analysis using a three-level model (i. e., participant, measurement, rater). This would allow inter-rater reliability as well as other model outcomes to be assessed simultaneously. We also acknowledge that the study was small in scale and limited to a single group of participants at our institution, thereby limiting the generalizability of our findings. Further research is necessary to replicate these findings across a broad range of participant groups, simulation environments and tasks.

In conclusion, the results of this study provide a proof-of-principle for future studies incorporating cognitive load measures in simulation-based assessment. Using the development of point-of-care ultrasonography skills as a model, we demonstrated that a combination of demographic and cognitive load measures can be used to predict performance in a simulated setting. The ability to maintain good performance with low cognitive load is an important marker of expertise. Performance assessments which include cognitive load can help differentiate between levels of expertise in simulation environments, and may thus serve as better predictors of skill transfer to clinical practice.

## Caption Electronic Supplementary Material




