# Supplementary material for: Cognitive load predicts point-of-care ultrasound simulator performance
Source: Perspect Med Educ. 2018 Jan 5;7(1):23–32. doi: 10.1007/s40037-017-0392-7 (PMC5807260; doi:10.1007/s40037-017-0392-7)
Supplement: Supplementary file 1 [file 40037_2017_392_MOESM1_ESM.docx]

**Appendix 1 - Tests of Individual Predictors**

All tables in this appendix report the following parameters: regression coefficients (B), standard errors (SE), degrees of freedom (df), *t*- and *p*-values, and 95% confidence intervals (CI) for estimates of fixed effects. Tables A-1, A-2, and A-3 additionally report covariance (V), SE and Wald *Z* and *p*-values for estimates of covariance.

*Table A-1. Parameter estimates for marginal model with ultrasound image acquisition as the dependent variable and level of training as the predictor variable.*

| **Fixed effect** | **B (SE)** | **df** | ***t*-value** | ***p*-value** | **95% CI** |
| --- | --- | --- | --- | --- | --- |
| Intercept | .92 (.16) | 114 | 5.67 | < .01 | .60 to 1.25 |
| Level of training^1^ | .22 (.08) | 114 | 2.84 | .01 | .07 to .37 |
|  |  | | | | |
| **Covariance parameter** | **V (SE)** | **Wald *Z*** | ***p*-value** |  |  |
| Repeated measures | .56 (.07) | 7.55 | < .01 |  |  |

Note: ^1^ range 0 (med student) to 3 (staff)

*Table A-2. Parameter estimates for marginal model with ultrasound image acquisition as the dependent variable and prior ultrasound training as the predictor variable.*

| **Fixed effect** | **B (SE)** | **df** | ***t*-value** | ***p*-value** | **95% CI** |
| --- | --- | --- | --- | --- | --- |
| Intercept | .74 (.20) | 114 | 3.69 | < .01 | .34 to 1.13 |
| Prior ultrasound training^1^ | .33 (.10) | 114 | 3.23 | < .01 | .13 to .53 |
|  |  | | | | |
| **Covariance parameter** | **V (SE)** | **Wald *Z*** | ***p*-value** |  |  |
| Repeated measures | .55 (.07) | 7.55 | < .01 |  |  |

Note: ^1^ range 0 (none) to 3 (extensive)

*Table A-3. Parameter estimates for marginal model with ultrasound image acquisition as the dependent variable and cognitive load (Paas scale rating) as the predictor variable.*

| **Fixed effect** | **B (SE)** | **df** | ***t*-value** | ***p*-value** | **95% CI** |
| --- | --- | --- | --- | --- | --- |
| Intercept | 2.00 (.16) | 114 | 12.88 | < .01 | 1.70 to 2.31 |
| Cognitive load^1^ | -.16 (.03) | 114 | -4.67 | < .01 | -.23 to -.09 |
|  |  | | | | |
| **Covariance parameter** | **V (SE)** | **Wald *Z*** | ***p*-value** |  |  |
| Repeated measures | .50 (.07) | 7.55 | < .01 |  |  |

Note: ^1^ range 1 to 9

*Table A-4. Parameter estimates for random intercept models with Paas scale rating as the dependent variable.*

| **Cognitive load measure** | **Intercept** | | | | | **Fixed effect of parameter** | | | | |
| --- | --- | --- | --- | --- | --- | --- | --- | --- | --- | --- |
|  | **B (SE)** | **df** | ***t*-value** | ***p*-value** | **95% CI** | **B** | **df** | ***t*-value** | ***p*-value** | **95% CI** |
| NASA-TLX^1^ | 1.48 (.22) | 65.78 | 6.65 | < .001 | 1.04 to 1.93 | .067 (.004) | 112.89 | 16.93 | < .001 | .060 to .075 |
| Pupil diameter range (mm) | 2.77 (.36) | 53.85 | 7.60 | < .001 | 2.04 to 3.50 | 1.20 (.25) | 92.33 | 4.90 | < .001 | .71 to 1.69 |
| Pupil diameter mean (mm) | 5.76 (1.83) | 35.34 | 3.15 | .003 | 2.05 to 9.47 | -0.34 (.37) | 35.62 | -.91 | .37 | -1.10 to .42 |
| Blink rate^2^ | 3.53 (.42) | 64.17 | 8.41 | < .001 | 2.69 to 4.37 | 2.84 (1.59) | 104.93 | 1.78 | .08 | -.32 to 6.00 |
| Total scanning time (s) | 2.88 (.29) | 43.89 | 10.02 | < .001 | 2.30 to 3.46 | .02 (.003) | 87.84 | 8.82 | < .001 | .018 to .029 |
| Gaze shift rate (gaze shifts/min) | 5.23 (.35) | 69.26 | 14.91 | < .001 | 4.53 to 5.92 | -.14 (.03) | 97.94 | -4.65 | < .001 | -.19 to -.08 |

Notes: Each row represents a different model; ^1^ range 0 to 120; ^2^ range 0 to 1.
